# Ultrafast Investigation of Multiple Strong Coupling System Based on Monolayer MoS_2_-Ag Nanodisk Arrays

**DOI:** 10.3390/nano16050339

**Published:** 2026-03-09

**Authors:** Jia Zhang, Yuxuan Chen, Leyi Zhao, Menghan Xu, Hai Wang

**Affiliations:** 1 State Key Laboratory of Integrated Optoelectronics, JLU Region, College of Electronic Science and Engineering, Jilin University, 2699 Qianjin Street, Changchun 130012, China; zhangj23@mails.jlu.edu.cn (J.Z.); xumenghan2001@gmail.com (M.X.); 2 Department of Applied Physics, The Hong Kong Polytechnic University, Kowloon, Hong Kong 999077, China; yuxuan66.chen@connect.polyu.hk

**Keywords:** strong coupling, surface plasmon polaritons, transition metal dichalcogenides

## Abstract

A multiple strong coupling system comprising monolayer MoS_2_ and Ag nanodisk (Ag-ND) arrays is investigated using transient absorption (TA) spectroscopy. By tuning the diameter and period of the Ag-NDs arrays, the surface plasmon polariton (SPP) resonances are made to simultaneously overlap with the A (~660 nm) and B (~608 nm) excitons of monolayer MoS_2_. As a result, three distinct negative ground-state bleaching (GSB) peaks, corresponding to the upper (UP), middle (MP), and lower (LP) hybrid polariton states, were observed in the TA spectra. This confirms that a multiple strong coupling regime was achieved with both the A and B excitons of monolayer MoS_2_ and SPPs modes, which was also highlighted by the anti-crossing behavior across varied Ag-NDs arrays parameters. Finally, by adding an insulating spacer layer of Al_2_O_3_ film, the coupling strength can be modulated from a strong coupling regime to a weak coupling regime. These results reveal a multi-exciton–plasmon strong coupling system and establish a versatile platform for ultrathin polaritonic devices, including polariton lasers and all-optical switches.

## 1. Introduction

In recent years, strong light–matter coupling with 2D materials has drawn great interest and application potential [1]. Compared to bulk materials, 2D materials with reduced dielectric screening and strong quantum confinement have become one of the most preferred candidates for the research of strong coupling, which is an extreme form of light–matter interaction [2]. In a strong coupling regime, the energy can be exchanged back and forth between light and matter, and the rate of the exchange is faster than any decoherence process, leading to the formation of light–matter hybrid states [3,4,5,6]. With the advantages of both light and matter, strong coupling opens up a very good opportunity to realize nanoplasmon devices [7,8,9,10,11,12,13,14,15], such as polariton lasers [16,17], all-optical polariton transistors [18,19], supercapacitors [20], and photoelectrochemical systems [21,22,23,24].

Monolayer transition metal dichalcogenides (TMDCs), as a typical type of 2D materials, possess unique optical and mechanical properties [25,26,27], such as high absorption efficiency, and turn to direct band gap semiconductors when reduced to a monolayer; more exciting opportunities for realizing ultrathin and flexible devices are offered [28]. Monolayer TMDCs possess two key attributes that make them ideal for forming light–matter strong coupling systems with surface plasmon polaritons (SPPs) [29,30,31]. First, their weak dielectric screening and strong quantum confinement [32,33], which result in exceptionally high exciton binding energies (several hundreds of meV) [34,35], thereby ensure the stability of excitons even at room temperature [36]. Second, their atomic-level thickness means all constituent atoms are exposed to the environment, rendering them highly susceptible to external modulation [37,38]. In monolayer TMDCs, strong spin–orbit coupling induces an energy splitting at the valence band maximum, giving rise to two distinct exciton species: the A exciton and the B exciton [39]. Due to its higher absorption efficiency, most studies on strong coupling have focused primarily on the A exciton. However, because the A and B excitons are intrinsically coupled via spin–orbit interaction, monolayer TMDCs offer an excellent platform for exploring multiple strong coupling regimes [36,40,41]. To date, such multiple strong coupling systems remain largely unexplored; in particular, the dynamics of TMDCs-SPPs coupled systems have not yet been reported. In this work, we demonstrate that multiple strong coupling systems can be formed with monolayer MoS_2_ and SPPs supported by Ag nanodisks (Ag-NDs) by transient absorption (TA) spectroscopy experiments. Furthermore, we demonstrate that the coupling strength can be tuned by controlling the distance between the monolayer MoS_2_ and Ag-NDs.

## 2. Materials and Methods

### 2.1. Sample Preparation

The Ag-ND arrays with different periods (p) and diameters (d) were fabricated on sapphire substrates by e-beam lithography (EBL). Positive resist (950 PMMA AR-P 672.045) was first spin-coated on the sapphire substrate and annealed at 150 °C for 3 min. Then, the sample was patterned by EBL machine and developed in a conventional solution of MIBK:IPA = 1:3 for 3 min followed by isopropanol washing for 1 min. Afterward, 50 nm Ag was deposited by electron beam deposition (evaporation rate 2 Å/s). After PMMA was soaked off with acetone, the fabrication of Ag NDs arrays was completed. For the samples with the Al_2_O_3_ spacer layer, the spacer layer was deposited using electron beam evaporation. The deposition rate was controlled at 0.3–0.5 Å/s, and the evaporation source material was high-purity Al_2_O_3_ granules (99.99% purity) purchased from Zhongjinyan Co., Ltd., Beijing, China. The commercial monolayer MoS_2_ samples used in this study were purchased from Shenzhen SixCarbon Technology Co., Ltd., Shenzhen, China and were prepared by chemical vapor deposition (CVD). The samples were grown directly on SiO_2_/Si substrates, exhibiting discretely distributed triangular single-crystalline grains with edge lengths of approximately 10–20 μm. An optical microscopy image is shown in Figure S3 in the Supporting Information.

### 2.2. Wet Transfer Method of Monolayer WS_2_ onto the Gratings

The transfer process referred to Lu’s work [42]. First, 1.5 g of PVP (Alfa Aesar, Haverhill, MA, USA, average MW 58 000), 1.5 mL of NVP (J&K, Beijing, China, 99.5%), 0.75 mL of deionized water, and 7 mL of ethanol were mixed in a vial for 10 min at room temperature. The solution was spin-coated on the triangular monolayer of MoS_2_ flakes adhered to the SiO_2_/Si substrate at 2000 rpm for 1 min and then baked at 70 °C for 1 min. After that, a 9 wt % PVA film (Alfa Aesar, Haverhill, MA, USA, 98–99% hydrolyzed, high molecular weight) was spin-coated on top of the sample at 2000 rpm for 1 min. The PVA/NVP film with triangular monolayer MoS_2_ flakes was peeled off from the SiO_2_/Si substrate and then attached to the Ag-grating substrate through electrostatic force. Finally, the MoS_2_-Ag NDs arrays were washed in deionized water for 1 h to remove the PVA/NVP film. The process is shown schematically in Figure S2.

### 2.3. Optical Measurements

The femtosecond transient absorption (TA) experiments were performed using a 100 fs laser pump-probe setup. A mode-locked titanium sapphire laser/amplifier system (Solstice, Spectra-Physics, Milpitas, CA, USA) was used. The output was split into two laser beams. The stronger part was sent to an optical parametric amplifier (TOPAS C, Spectra-Physics Milpitas, CA, USA) to generate a pump laser at 560 nm. The other beam with lower energy was focused on a 5 mm thick sapphire crystal to generate a broad white-light continuum (from 450 nm to 800 nm) as a probe beam. The pump and probe lasers were set as collinear by a beam splitter cube and focused on the same position of the sample at normal incidence. The diameter of the probe spot is about 5 μm under optimized focusing, while the pump spot is a little larger due to the chromatic aberrations. The delay time between the pump and the probe pulses was controlled by a motorized mechanical delay stage (DL325, Newport, Irvine, CA, USA). Finally, the reflected probe light from the sample was collected by the same objective and sent to a highly sensitive spectrometer (Avantes-ULS2048CL-EVO, Avantes, Apeldoorn, The Netherlands). The group velocity dispersion of the whole experimental system was compensated by a chirp program.

## 3. Results and Discussion

### 3.1. Sample Fabrication and Steady-State Measurements

**Figure 1:**
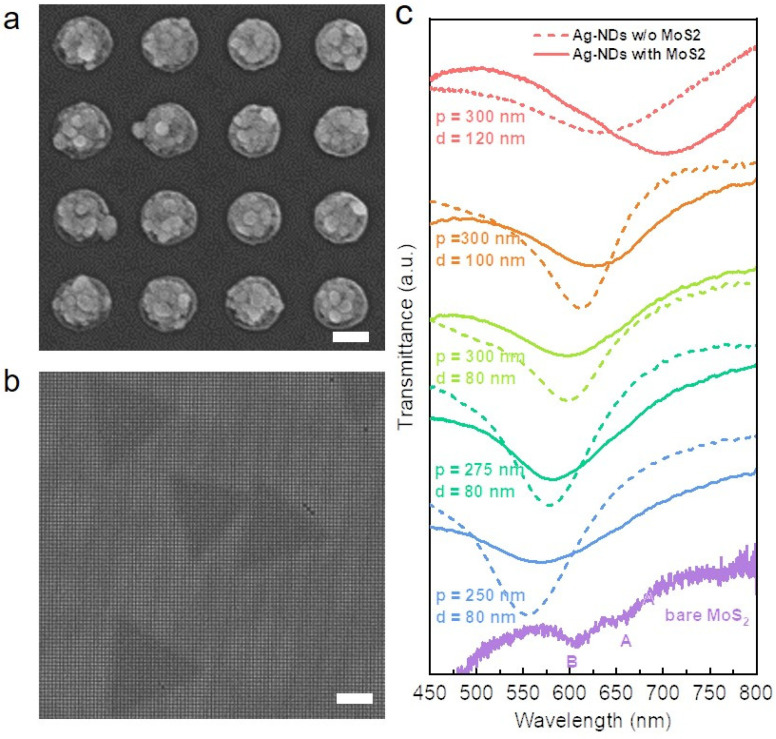
(**a**) The SEM image of bare Ag-NDs; the scale bar is 100 nm. (**b**) The SEM image of bare Ag-NDs with monolayer MoS_2_; the scale bar is 5  μm. (**c**) The offset transmission spectra of Ag-NDs (dashed line) and monolayer MoS_2_-Ag-NDs (solid line). Here, p and d represent the period and diameter of the Ag-NDs arrays, respectively. The purple solid line is the transmission spectrum of the monolayer MoS_2_.

The Ag-ND arrays with different periods (p) and diameters (d) are fabricated on a sapphire substrate by electron beam lithography (EBL) (see Methods in the Supporting Information (SI)). The SEM image of the Ag-ND arrays is shown in Figure 1a. The height of the Ag-NDs (h) is 50 nm. When the diameter of Ag-ND (d) is 80 nm, their periods are selected as 250 nm, 275 nm, and 300 nm, respectively. When the period of Ag-ND arrays is 300 nm, their diameters are 80 nm, 100 nm, and 120 nm, respectively. The transmission spectra of the Ag-ND arrays are shown by the dashed lines in Figure 1c. When the diameter of the Ag-NDs is 80 nm, the transmission peak is red-shifted from 550 nm to 578 nm and 600 nm for the arrays with periods of 250 nm, 275 nm, and 300 nm, respectively. When the period of the Ag-ND arrays is 300 nm, the diameter is varied between 80 nm, 100 nm, and 120 nm. The corresponding transmission peaks are at 600 nm, 616 nm, and 635 nm, respectively. As the diameter of the Ag nanodisk increases, the transmission peaks of Ag-ND arrays also show a red shift. Therefore, by adjusting the period and diameter of the Ag-ND arrays, the transmission peaks of the SPPs can be tuned from 550 nm to 635 nm. In addition, the broad transmission peaks can cover both the A and B excitons of monolayer MoS_2_ simultaneously, which is suitable for forming multiple strong coupling systems.

Then, triangular monolayer MoS_2_ flakes are transferred to the Ag-ND arrays by a wetting transfer technology (see Methods (Figure S2) in the Supporting Information). The SEM image of the Ag-NDs/MoS_2_ are shown in Figure 1b. The steady-state spectra of these samples are studied as shown in Figure 1c. As the reference sample, the transmission spectrum of monolayer MoS_2_ is shown with a purple solid line. Due to the weak absorption of monolayer MoS_2_, the measured transmission spectrum of monolayer MoS_2_ is amplified five times to enhance the visibility of the transmission peaks. The two transmission peaks at 660 nm and 608 nm correspond to the absorption of the A exciton and B exciton in monolayer MoS_2,_ respectively. The transmission spectra of the monolayer MoS_2_-Ag NDs samples are shown by the solid line in Figure 1c. As can be seen, the transmission peak of the monolayer MoS_2_-Ag-ND arrays is significantly red-shifted compared to that of the pure Ag-ND arrays. However, due to the absorption intensity of monolayer MoS_2_ being relatively weak compared to the resonance absorption of the SPPs of Ag-ND arrays, there is no obvious splitting in the steady-state transmission spectra. Hence, it is difficult to determine whether multiple strong coupling systems are formed. To qualitatively describe the multiple strong coupling between the A/B excitons and the SPP mode, we use a three-level Jaynes–Cummings-type Hamiltonian including the A exciton $\omega_{A}$, B exciton $\omega_{B}$ and a single SPP resonance $\omega_{spp}$ [12,40].

$$H=\left(\begin{array}{ccc} \omega_{A} & J_{AB} & g_{A}\\ J_{AB} & \omega_{B} & g_{B}\\ g_{A} & g_{B} & \omega_{spp} \end{array}\right).$$

By diagonalizing the 3 × 3 Hamiltonian with realistic exciton energies and coupling strengths $g_{A}$ and $g_{B}$, as shown in Figure S4, we obtain three hybridized polariton branches (UP, MP and LP). We neglected the direct coupling $J_{AB}$ between excitons A and B originating from spin–orbit and Coulomb many-body effects. Next, to gain insight into the formation of the coupling systems, TA spectroscopy experiments were further conducted on the MoS_2_-Ag-NDs arrays.

### 3.2. Transient Absorption Experiments

**Figure 2:**
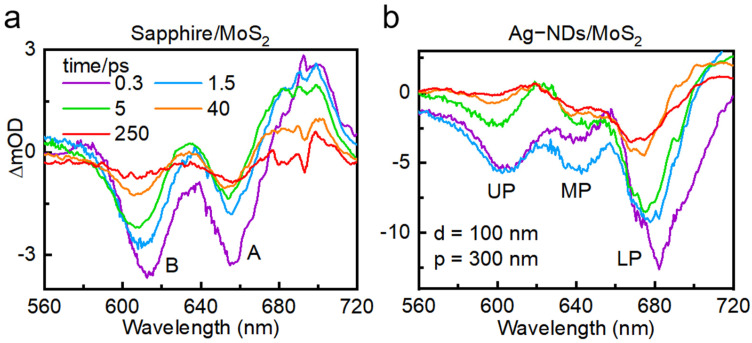
The TA spectra of (**a**) bare monolayer MoS_2_ and (**b**) monolayer MoS_2_-Ag-ND arrays at different delay times (0.3, 1.5, 5, 40, and 250 ps).

In TA experiments, more information about the excited state in the hybrid systems can be reflected by the variation in the optical density (∆OD = OD_pump_ − OD_without pump_). TA experiments (see Methods for details) on the monolayer MoS_2_-Ag-ND arrays were performed by a 690 nm pump laser pulse. As a reference, the TA spectra of monolayer MoS_2_ on the sapphire substrate are illustrated in Figure 2a. The dominated negative ground-state bleaching (GSB) signal at 615 nm and 666 nm corresponds to the A and B excitons of monolayer MoS_2_, respectively. The TA spectra of the monolayer MoS_2_-Ag nanodisk arrays with different periods and diameters are shown in Figure S1. The coupled samples exhibited completely different properties if compared with the reference one. The TA spectra of the MoS_2_-Ag NDs arrays with d = 100 nm and p = 300 nm are shown in Figure 2b as a representative. As can be seen, three distinct negative signals appear at 590 nm, 630 nm, and 680 nm, which can be assigned to the up, middle, and lower hybrid states (UP, MP, and LP) of the coupling system. Meanwhile, it can be observed that the splitting value of the coupled sample is larger than the linewidths of the A exciton and B exciton of MoS_2_, which is typical evidence of multiple strong coupling (see Figure S5 in the Supporting Information). Notably, under resonant excitation, clear negative ground-state bleaching peaks were observed in the TA spectra, with no obvious positive signals. This phenomenon further confirms that thermal effects and non-coherent processes have been effectively suppressed, and the dynamic behavior of the hybrid states primarily originates from the coherent coupling processes [43].

**Figure 3:**
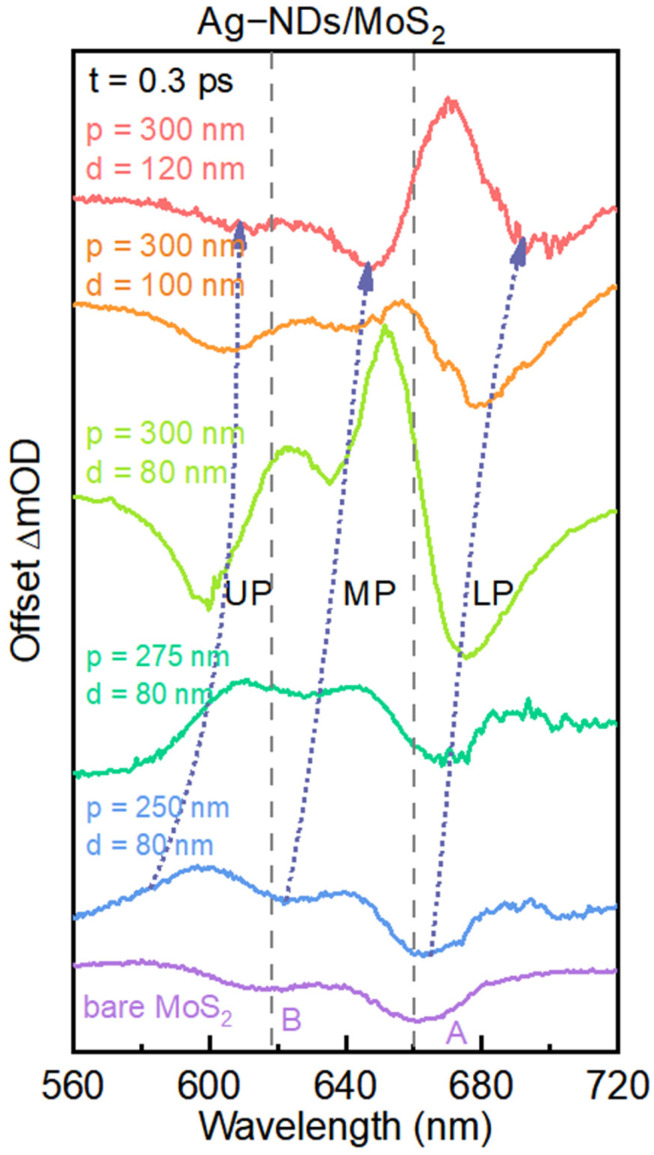
TA spectra of bare monolayer MoS_2_ (the purple solid) line and monolayer MoS_2_-Ag-ND arrays with different parameters at 0.3 ps.

**Figure 4:**
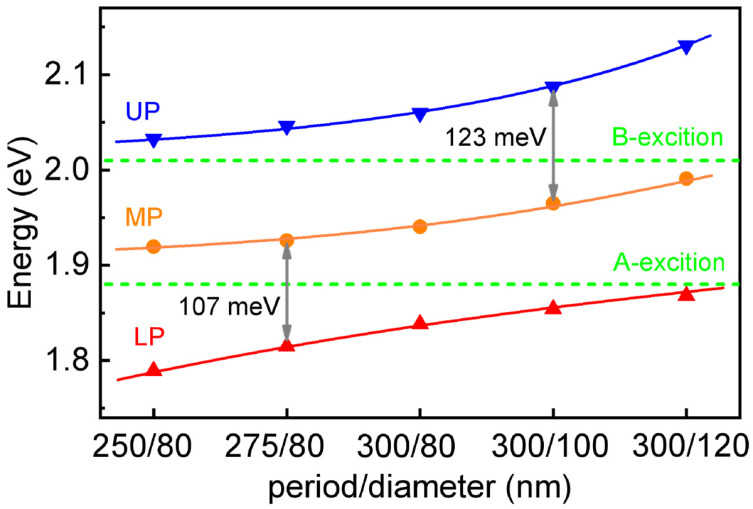
The energy dispersion of the UP, MP, and LP in the multiple strong coupling systems of monolayer MoS_2_-Ag-ND arrays; the green dashed lines represent the energies of the A exciton and B exciton of MoS_2_.

Furthermore, to provide more sufficient evidence of the multiple strong coupling regime, the initial TA spectra at 0.3 ps for the samples with different lattice periods and diameters are extracted and compared in Figure 3. The solid purple line represents the TA spectrum of monolayer MoS_2_ at 0.3 ps. The positions corresponding to the absorption peak of the A exciton and B exciton in monolayer MoS_2_ are reported with dashed gray lines. Then, it can be noted that, with the SPP resonances having red-shifted, the UP, MP, and LP also show a trend of red-shifting. Subsequently, we extracted the peak energies of the UP, MP, and LP from the TA spectra in Figure 3 and plotted them as a function of the structural parameters (diameter and period) of the Ag-ND arrays. As shown in Figure 4, with the SPP resonance energy systematically scanned across the A and B excitons of monolayer MoS_2_, the three hybrid branches (UP, MP, and LP) exhibit clear anti-crossing behavior, from which double vacuum Rabi splitting energies up to 107 meV and 123 meV can be observed. Thus, we further identify that a complex multiple strongly coupled system is formed between the A and B excitons in monolayer MoS_2_ and the SPP modes in the Ag-ND arrays.

**Figure 5:**
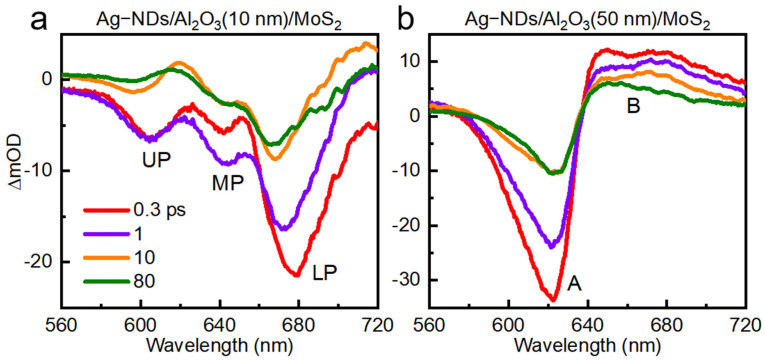
TA spectra of the monolayer MoS_2_-Ag-ND arrays (d = 100 nm, p = 300 nm) with (**a**) 10 nm and (**b**) 50 nm spacing at different delay times (0.3, 1, 10, and 80 ps).

More importantly, since the intensity of SPP decays exponentially in the direction perpendicular to the interface, we can adjust the coupling strength by controlling the distance between the monolayer MoS_2_ and Ag-ND arrays. Specifically, the spacing between the excitons in monolayer MoS_2_ and SPP modes can be controlled by depositing a layer of Al_2_O_3_ films with different thicknesses on the Ag-ND arrays. First, when the thickness of the space layer of Al_2_O_3_ films is 10 nm, the TA spectra of the monolayer MoS_2_-Ag-ND arrays (d = 100 nm, p = 300 nm) are shown in Figure 5a. As can be seen, three distinct GSB peaks can still be observed. Namely, under a 10 nm spacer, the A and B excitons of monolayer MoS_2_ and SPPs modes can still form a multiple strong coupling system.

**Figure 6:**
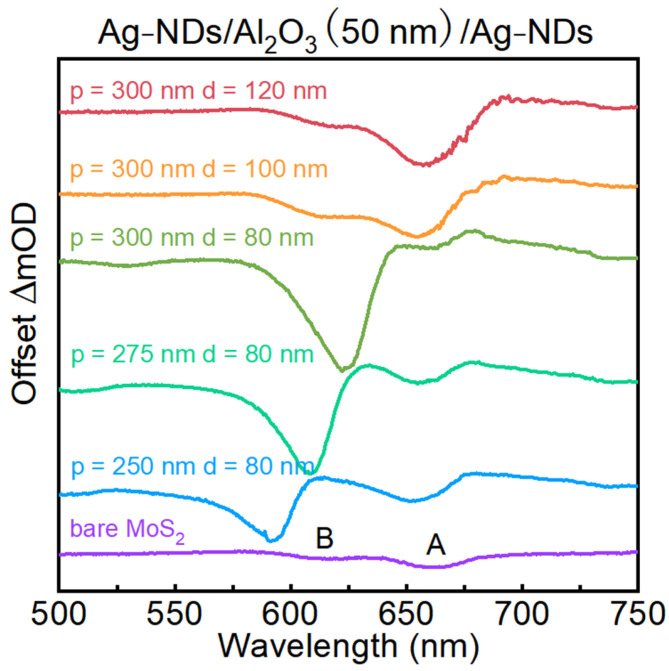
TA spectra of the monolayer MoS_2_-Ag-ND arrays with 50 nm spacing at 0.3 ps.

Then, when the thickness of the Al_2_O_3_ film is increased to 50 nm, the TA spectra of the monolayer MoS_2_-Ag-ND arrays with 50 nm spacing (d = 100 nm, p = 300 nm) are shown in Figure 5b. Unlike the TA spectra of the monolayer MoS_2_-Ag-ND arrays with 0 nm and 10 nm spacing, only two GSB peaks without splitting are observed in the TA spectra. This indicates that the energy level structure of excitons was not affected. In this case, as shown in Figure 6, the GSB signals of the A excitons or B excitons of monolayer MoS_2_ can be amplified when we tune the SPPs resonances to overlap with them. This can be attributed to the Purcell effect in the weak coupling regime. Therefore, by increasing the distance between the monolayer MoS_2_ and Ag-ND arrays, the multiple coupling system can be adjusted from a strong coupling regime to a weak coupling regime.

## 4. Conclusions

In summary, we have experimentally realized and characterized a multiple strong coupling system between the A and B excitons in monolayer MoS_2_ and the SPP modes in Ag-ND arrays. By using ultrafast TA spectroscopy, clear observation of three hybrid polariton branches (UP, MP, LP) with characteristic anti-crossing behavior confirms simultaneous strong coupling of both A and B excitons to the SPP mode. Furthermore, by precisely controlling the distance between the monolayer MoS_2_ and the Ag-NDs via Al_2_O_3_ spacers, we demonstrate that the hybrid system can be tuned from the strong coupling regime to the weak coupling regime. This distance-dependent control enables active modulation of coupling strength on demand. This work paves the way for the development of compact, flexible, and ultrafast polaritonic devices, including polariton lasers, optical switches, sensors, and potential quantum information components.

## Data Availability

Data available on request from the corresponding author.

## Supplementary Materials

The following supporting information can be downloaded at: https://www.mdpi.com/article/10.3390/nano16050339/s1, Figure S1. The TA spectra of bare MoS_2_ and monolayer MoS_2_-Ag-ND arrays at different delay times (0.3, 1.5, 5, 40, and 250 ps); Figure S2. The schematic diagram of the fabrication process of the Ag-NDs/MoS_2_ hybrid systems; Figure S3. Microscopy image of triangular MoS_2_ deposited on a Si/SiO2 substrate; Figure S4. Schematic diagram of the energy dispersion of UP, MP, and LP in monolayer MoS_2_-Ag ND arrays under different SPP resonance; Figure S5. Comparison of the TA spectra of bare monolayer MoS_2_ on sapphire and the MoS_2_-Ag-NDs (p = 300 nm, d = 100 nm) hybrid system at the delay time of 0.3 ps. Table S1. Comparison of representative multiple strong coupling systems based on TMDCs-metal structures [44,45,46,47].
