# Supplementary material for: Ultrafast Investigation of Multiple Strong Coupling System Based on Monolayer MoS2-Ag Nanodisk Arrays
Source: Nanomaterials (Basel). 2026 Mar 9;16(5):339. doi: 10.3390/nano16050339 (PMC12987320; doi:10.3390/nano16050339)
Supplement: Supplementary file 1 [file nanomaterials-16-00339-s001.zip › nanomaterials-4160120-supplementary.pdf]

Article

Ultrafast Investigation of Multiple Strong Coupling System Based on Monolayer MoS_2-Ag Nanodisk Arrays

Jia Zhang [1], Yuxuan Chen [2], Leyi Zhao [2,*], Menghan Xu [1] and Hai Wang [1,*]

1 State Key Laboratory of Integrated Optoelectronics, JLU Region, College of Electronic Science and Engineering, Jilin University, 2699 Qianjin Street, Changchun 130012, China; zhangj23@mails.jlu.edu.cn (J.Z.); xumenghan2001@gmail.com (M.X.)

2 Department of Applied Physics, The Hong Kong Polytechnic University, Kowloon, Hong Kong 999077, China; yuxuan66.chen@connect.polyu.hk

* Correspondence: leyi24.zhao@polyu.edu.hk (L.Z.); wanghai03@jlu.edu.cn (H.W.)

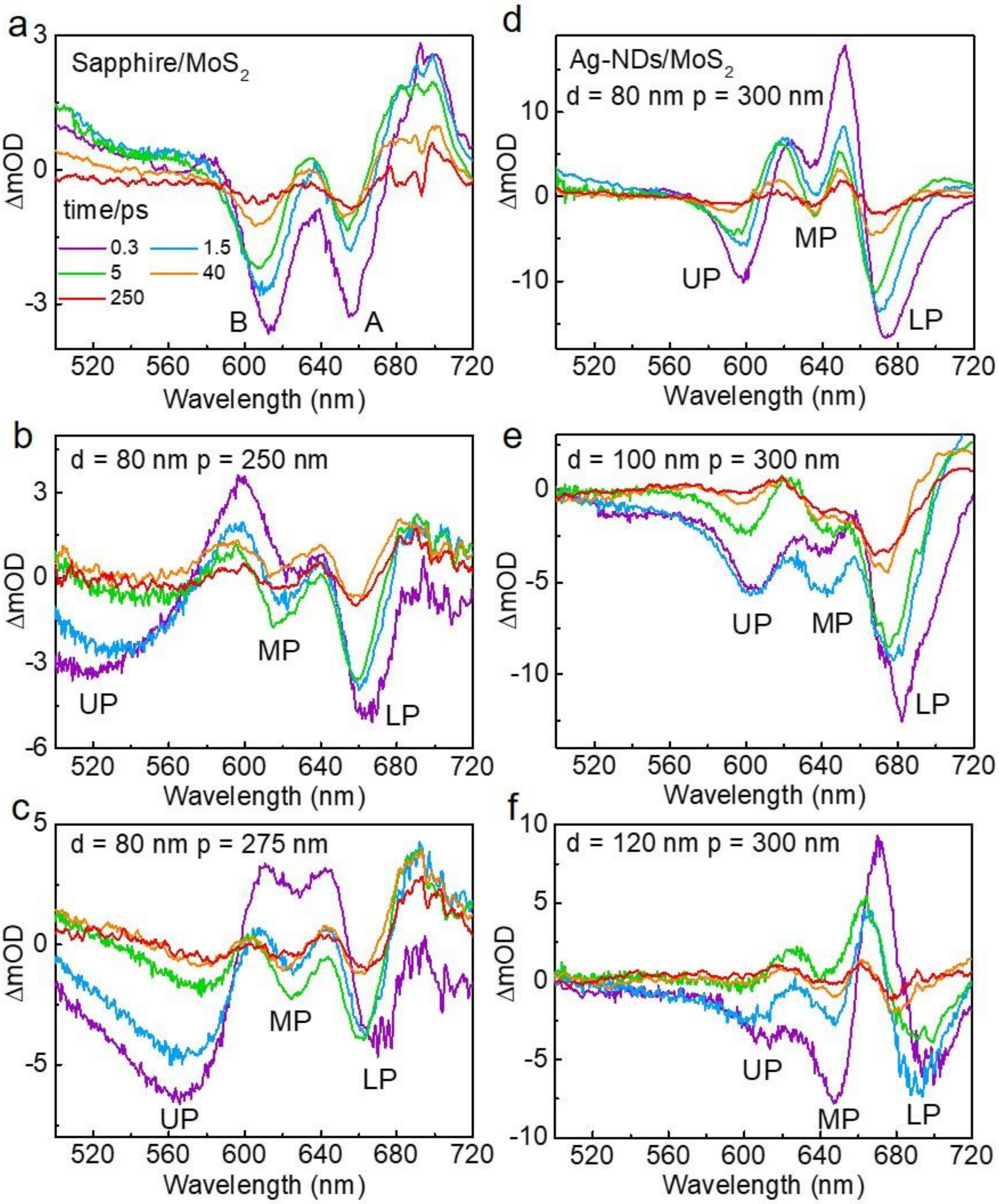

Figure S1. The TA spectra of bare MoS_2 and monolayer MoS_2-Ag-NDs arrays at different delay times (0.3, 1.5, 5, 40, and 250 ps).

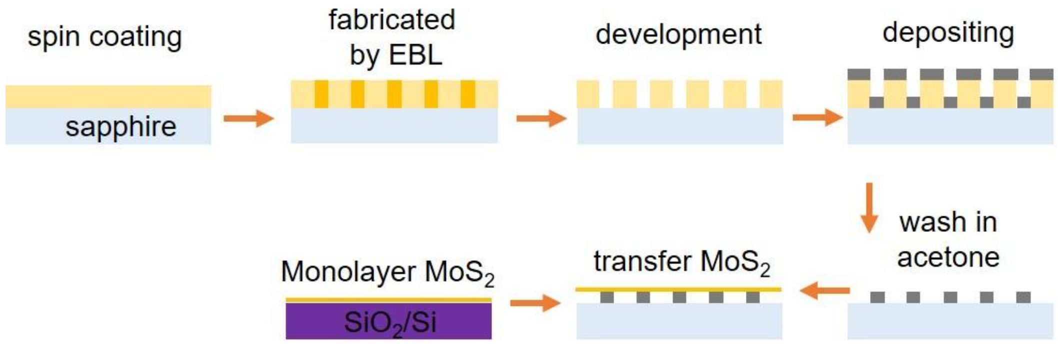

Figure S2. The schematic diagram of the fabrication process of the Ag-NDs/MoS_2 hybrid systems.

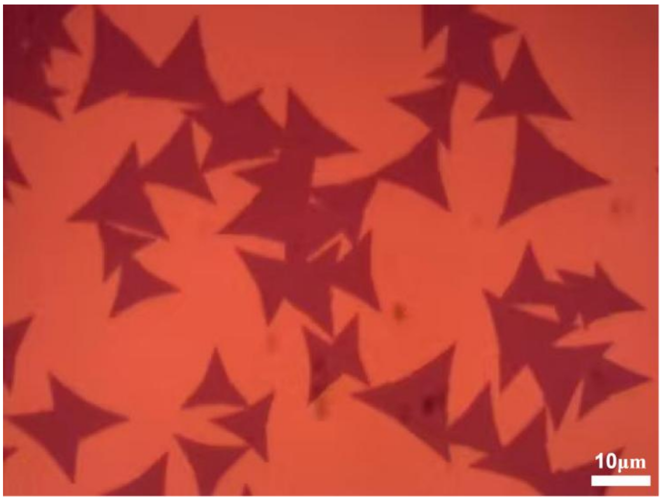

Figure S3. Microscopy image of triangular MoS_2 deposited on a Si/SiO_2 substrate.

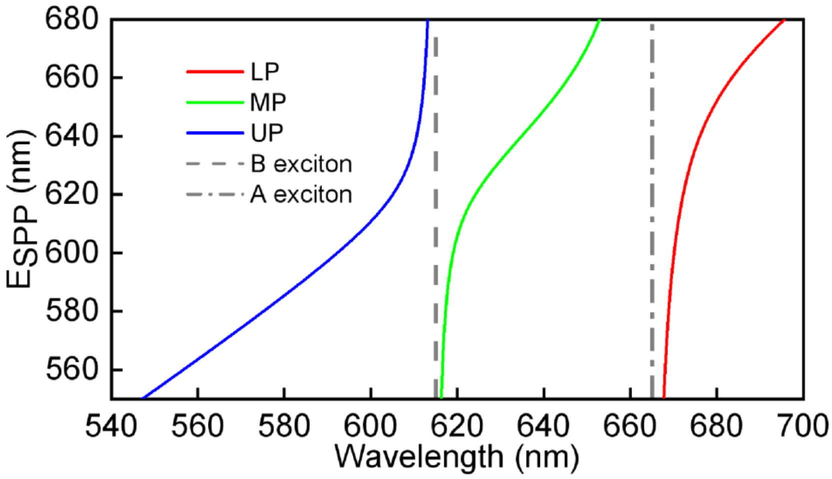

Figure S4. Schematic diagram of the energy dispersion of UP, MP, and LP in monolayer MoS_2-Ag NDs arrays under different SPP resonance.

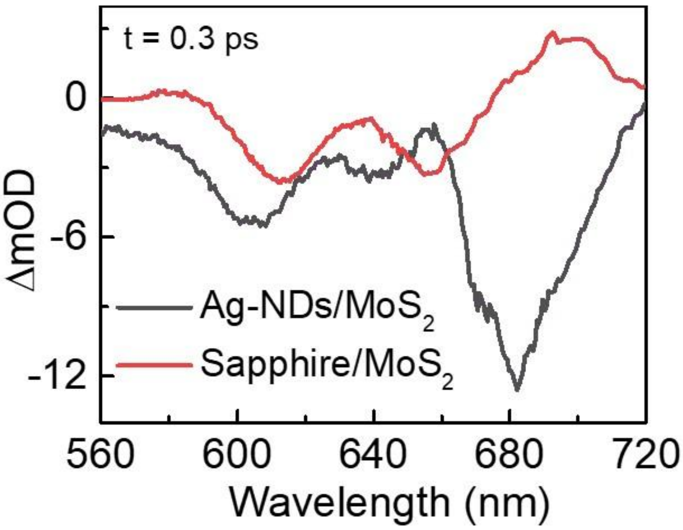

Figure S5. Comparison of the TA spectra of bare monolayer MoS_2 on sapphire and the MoS_2-Ag-NDs (p = 300 nm, d = 100 nm) hybrid system at the delay time of 0.3 ps.

Table S1. Comparison of representative multiple strong coupling systems based on TMDCs-metal structures.

Material System	Coupling Scheme	Characterization	Rabi Splitting	References
Few-Layer MoS_2	prism-excited surface plasmon polaritons and the A and B excitons of MoS_2	Steady-state characterization	A exciton: ~81 meV<br>B exciton: ~93 meV	1
Monolayer WS_2	TE-polarized waves supported by the Si_3N_4/Ag heterostructure and the A and B excitons in WS_2	Steady-state characterization	A exciton: ~149 meV<br>B exciton: ~147 meV	2
Ag-WS_2	plasmons, the A exciton, and optical microcavity photons in an Ag-WS_2 heterostructure	Steady-state characterization	~300 meV	3
Monolayer WS_2	localized surface plasmons in silver nanoprisms and excitons and charged trions in monolayer WS_2	Steady-state characterization	~150 meV	4
Monolayer MoS_2	surface plasmon polaritons excited by metallic structures and the A and B excitons in monolayer MoS_2	Transient characterization	A exciton: ~107 meV<br>B exciton: ~123 meV	This Work

Supplementary References

1. Rose, A.; Dunklin, J.; Zhang, H.; Merlo, J.; van de Lagemaat, J. Plasmon-Mediated Coherent Superposition of Discrete Excitons under Strong Exciton–Plasmon Coupling in Few-Layer MoS_2 at Room Temperature. *ACS Photonics* **2020**, 7, 1129-1134.
2. He, W.; Liu, S.; Liu, J.; Li, S.; Deng, F.; Liu, H.; Fan, H.; Dai, J.; Lan, S. Hybridization of the A- and B-Exciton in a WS_2 Monolayer Mediated by the Transverse Electric Polarized Wave Supported by a Si_3N_4/Ag Heterostructure. *ACS Appl. Nano Mater*. **2024**, 7, 19089-19100.
3. Li, B.; Zu, S.; Zhang, Z.; Zheng, L.; Jiang, Q.; Du, B.; Luo, Y.; Gong, Y.; Zhang, Y.; Lin, F.; Shen, B.; Zhu, X.; Ajayan, P.M.; Fang, Z. Large Rabi splitting obtained in Ag-WS_2 strong-coupling heterostructure with optical microcavity at room temperature. *OPTO-ELECTRON ADV* **2019**, *2*, 190008-190001.
4. Cuadra, J.; Baranov, D.G.; Wersäll, M.; Verre, R.; Antosiewicz, T.J.; Shegai, T. Observation of Tunable Charged Exciton Polaritons in Hybrid Monolayer WS_2−Plasmonic Nanoantenna System. *Nano Lett*. **2018**, *18*, 1777-1785.
